# Review of the Role of Ferroptosis in Testicular Function

**DOI:** 10.3390/nu14245268

**Published:** 2022-12-10

**Authors:** Xu Yang, Yunhe Chen, Wenxi Song, Tingyu Huang, Youshuang Wang, Zhong Chen, Fengjuan Chen, Yu Liu, Xuebing Wang, Yibao Jiang, Cong Zhang

**Affiliations:** 1 College of Veterinary Medicine, Henan Agricultural University, Zhengzhou 450002, China; 2 College of Animal Science and Technology, Henan Agricultural University, Zhengzhou 450002, China

**Keywords:** ferroptosis, testis, male reproductive function, iron

## Abstract

Iron is an important metal element involved in the regulation of male reproductive functions and has dual effects on testicular tissue. A moderate iron content is necessary to maintain testosterone synthesis and spermatogenesis. Iron overload can lead to male reproductive dysfunction by triggering testicular oxidative stress, lipid peroxidation, and even testicular ferroptosis. Ferroptosis is an iron-dependent form of cell death that is characterized by iron overload, lipid peroxidation, mitochondrial damage, and glutathione peroxidase depletion. This review summarizes the regulatory mechanism of ferroptosis and the research progress on testicular ferroptosis caused by endogenous and exogenous toxicants. The purpose of the present review is to provide a theoretical basis for the relationship between ferroptosis and male reproductive function. Some toxic substances or danger signals can cause male reproductive dysfunction by inducing testicular ferroptosis. It is crucial to deeply explore the testicular ferroptosis mechanism, which will help further elucidate the molecular mechanism of male reproductive dysfunction. It is worth noting that ferroptosis does not exist alone but rather coexists with other forms of cell death (such as apoptosis, necrosis, and autophagic death). Alleviating ferroptosis alone may not completely reverse male reproductive dysfunction caused by various risk factors.

## 1. Introduction

Iron is an essential trace element for humans and animals; 70% of iron is stored in red blood cells, 20% in hepatocytes, 5% in reticuloendothelial cells, 3% in myocytes, and 2% in other cells [1]. Iron is an essential ingredient for oxygen transport and exchange and is a key component of many intracellular enzymes, including myoglobin, hemoglobin, cytochrome p450, iron-sulfur proteins, and the mitochondrial electron transport chain [2]. Iron plays a two-way role in regulating redox reactions in cells. Iron can aggravate the generation of reactive oxygen species (ROS) using the Fenton reaction (Fe^2+^ + H_2_O_2_ →Fe^3+^ +·OH + OH^−^) and Haber–Weiss reaction (Fe^3+^ + O_2_^·−^→Fe^2+^ + O_2_; Fe^2+^ + H_2_O_2_→Fe^3+^ +·OH + OH^−^), thereby increasing cellular oxidative stress. Furthermore, iron is the bioactivity center of antioxidant enzymes (catalase and superoxide dismutase) [3]. Iron homeostasis also has a very important effect on the body’s normal physiological metabolism [4]. Significant iron deficiency can directly block erythropoiesis, eventually resulting in anemia [5]. Iron deficiency can also induce digestive dysfunction and mental retardation. In contrast, iron overload is often seen as a potential risk factor for hemochromatosis and is also a pathogenic factor of neurodegenerative, liver, and kidney diseases [1]. Given the role of iron in health, maintenance of iron homeostasis is essential for normal life activities.

## 2. The Physiological Role of Iron in the Male Reproductive System

Iron plays an important role in spermatogenesis and testosterone synthesis [6]. Abnormal testosterone synthesis and impaired spermatogenesis are the main manifestations of male reproductive dysfunction. Transferrin (Tf) is an iron-binding protein that can bind with iron in spermatogenic cells in different developmental stages of the seminiferous tubule [7]. Sertoli cells transmit iron in the form of apical secretion to spermatogenic cells in different developmental stages on the germinal epithelium of seminiferous tubules and play a key role in promoting the growth, maturation, and release of germ cells [8]. Sertoli cell function defects inhibit Tf secretion and lead to spermatogenesis disorders, which affect male fertility. The Tf level in testicular tissue is a specific biomarker reflecting the function of Sertoli cells [9].

Severe iron deficiency in the body will directly lead to anemia [10], which creates a hypoxic environment for the testis. In this situation, the partial pressure of oxygen in the testis tissue is relatively low, the rate of oxygen diffusion is slow, and the ability to increase total blood flow is reduced. However, continuous spermatogenesis requires a considerable amount of oxygen consumption. Therefore, males suffering from iron-deficiency anemia may have poor semen parameters [11]. Iron deficiency increases testicular oxidative stress and decreases antioxidant enzyme activities, leading to lower serum testosterone and poorer spermatogenesis. A previous study reported that iron-deficient diets inhibited antioxidase (such as glutathione peroxidase and catalase) activity and hydroxysteroid dehydrogenase expression while enhancing cleaved caspase 8 and caspase 3 expression in rats [6]. Interestingly, testicular iron overload can also lead to the impairment of testicular function [12] since iron overload can cause spermatogenesis disorders and Leydig cell dysfunction [13]. Sperm cell membranes are also rich in polyunsaturated fatty acids, which are highly vulnerable to ROS attack, during which they undergo lipid peroxidation [14]. It is foreseeable that iron overload promotes ROS production through the Fenton reaction and can significantly impair male semen parameters through the oxidized sperm cell membrane [15]. Angélica et al., through in vitro spermatozoa culture experiments, found that ferrous iron/ascorbate (100 μM/150 μM) reduced spermatozoa motility and viability, decreased the mitochondrial membrane potential of spermatozoa, increased intracellular ROS generation, and impaired the fertilization capability of spermatozoa [16].

## 3. The Mechanism of Ferroptosis

**Figure 1:**
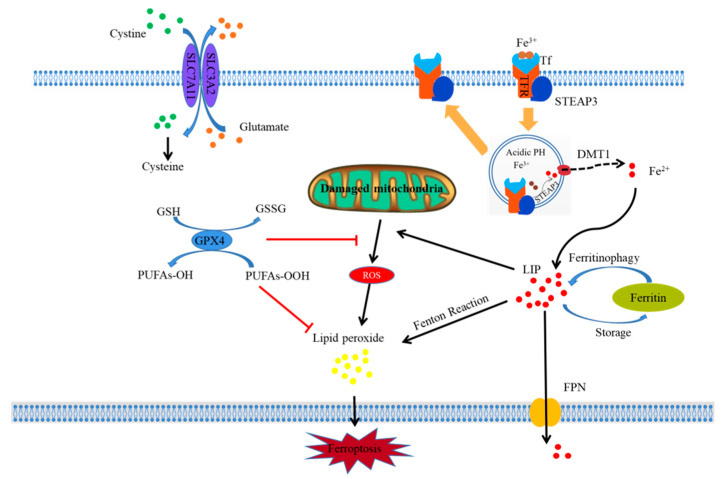
Schematic diagram illustrating the proposed mechanism of ferroptosis. Figure legend: The occurrence and regulatory mechanisms of ferroptosis in a cell. Fe^3+^, which is indispensable to ferroptosis, can bind with Tf to form a Tf–Fe^3+^ complex and is internalized in cells by TFR using endocytosis. Then, Fe^3+^ is dissociated from Tf in an acidic environment and reduced to Fe^2+^ by STEAP3. Subsequently, Fe^2+^ is released from the endosome into LIP in the cytoplasm via DMT1. Free Fe^2+^ further results in lipid peroxide through the Fenton reaction, which ultimately triggers the occurrence of ferroptosis. In addition, some ferroptosis inducers can inhibit system xc- and impede the uptake of cystine by cells, thus leading to a decline in intracellular cysteine and a subsequent reduction in GSH, which requires cysteine for its synthesis; this ultimately results in a decline in the anti-oxidative ability of cells. As a key component in ferroptosis, GPx4 can bind with GSH and suppress cellular lipid peroxides to prevent cellular ferroptosis. Meanwhile, some substances can directly suppress GPx4 to induce ferroptosis. Mitochondria are the most important organelle involved in ferroptosis, which are the main sites for the generation of ROS and release of ferroptosis-inducing lipid peroxides, especially when they are damaged.

Ferroptosis, a novel type of cell death, was discovered and reported by Dixon in 2012. The first evidence of ferroptosis was found in Ras-mutant tumor cells treated with erastin [17]. As a form of regulated cell death, ferroptosis is genetically, morphologically, and biochemically distinct from apoptosis, autophagy, necrosis, and necroptosis [18]. Ferroptosis is a type of iron-dependent cell death that mainly manifests as intracellular iron accumulation, lipid peroxidation, and mitochondrial damage. A simple mechanistic diagram of ferroptosis is shown in Figure 1.

### 3.1. Iron Overload and Ferroptosis

Intracellular iron homeostasis depends on the balance of iron uptake, storage, and outflow. Increasing iron absorption, reducing iron storage, and limiting iron outflow lead to iron overload and iron homeostasis imbalance, which are prerequisites for initiating ferroptosis. Tf is mainly responsible for the uptake of iron. When bound to extracellular Fe^3+^, the Tf–Fe^3+^ complex binds to the transferrin receptor (TfR), is internalized by endocytosis, and enters the cell [2]. In the cell, Fe^3+^ is dissociated from Tf in an acidic environment, and Fe^3+^ is reduced to Fe^2+^ by metal reductase 3 [19,20]. Subsequently, Fe^2+^ is released from the endosome into the labile iron pool (LIP) in the cytoplasm via divalent metal transporter 1 (DMT1).

Excess iron is stored in ferritin, which is the main intracellular iron storage protein complex, composed of ferritin light chain (FTL) and ferritin heavy chain (FTH) [21,22,23,24]. Iron is stored in the form of ferritin, and autophagic degradation of ferritin, also called ferritinophagy, leads to an increase in the intracellular free iron concentration. Free iron further results in the accumulation of intracellular ROS through the Fenton reaction, which ultimately triggers the occurrence of ferroptosis [25]. To a certain extent, ferroptosis is associated with an autophagic cell death process [26]. In addition, ferroportin (FPN) exports iron out of the cell as the sole iron outflow transporter in the plasma membrane. The low expression of FPN also reduces iron excretion from cells, resulting in intracellular iron accumulation. Currently, the specific mechanism of ferroptosis is still under debate, but it is clear that iron overload is a crucial event for the occurrence of ferroptosis.

### 3.2. Lipid Peroxidation and Ferroptosis

The accumulation of iron and lipid peroxide products (LPO) within cells is the basis for ferroptosis. One of the key triggers for ferroptosis is Fe^2+^/Fe^3+^, which is involved in the formation of ROS through enzymatic or nonenzymatic reactions [27]. Iron plays a key role in catalyzing the production of oxygen radicals and initiating the chain propagation of LPO in the ferroptosis process [28]. During electron transfer, oxygen receives electrons to form hydrogen peroxide (H_2_O_2_), Fe^2+^ and H_2_O_2_ reactivate hydroxyl radicals produced by Fenton reactions, and lipid peroxidation is initiated in synergy with polyunsaturated fatty acids (PUFAs) on the phospholipid cell membrane [17]. If free divalent iron increases for various reasons, excess Fe^2+^ leads to the accumulation of lipid ROS through the Fenton reaction, which leads to ferroptosis [11]. Lipid peroxidation is the ultimate executor of ferroptosis and is considered a landmark event of ferroptosis [29].

### 3.3. Glutathione Peroxidase Depletion and Ferroptosis

Depletion of the glutathione (GSH)/glutathione peroxidase 4 (Gpx4) system is a typical biochemical reaction of ferroptosis. The excessive oxidation reaction of Fe^2+^ with peroxidation groups and the harmful products produced by the oxidation reaction can impair the integrity and stability of cell membranes [30], but this reaction can be inhibited by iron chelators or properly removed by the GSH/Gpx4 system [31,32]. Under normal circumstances, LPO is converted into lipid alcohols, and GSH is oxidized to GSSG by Gpx4 catalysis, preventing ROS accumulation and maintaining cellular redox homeostasis [33]. However, when the intracellular GSH/Gpx4 system is depleted, Gpx4 is insufficient to promote GSH to convert LPO to water or corresponding lipid alcohols, which ultimately leads to ferroptosis [34].

GSH synthesis is based on cysteine, which is reduced from cystine. Cystine entry into cells is regulated by the cystine/glutamate exchange transporter (system xc-), which is an important intracellular antioxidant system on the cell membrane [27,35]. Inhibiting system xc- decreases the intracellular GSH level and impairs Gpx4 function. System xc- consists of a 12-pass transmembrane protein transporter (SLC7A11) and a single-pass transmembrane regulatory protein (SLC3A2). SLC3A2 acts as a chaperone protein to support the function of SLC7A11. SLC7A11 is an amino acid transporter and the main functional subunit of system xc-. SLC7A11 belongs to the heterodimeric amino acid transport family, which is responsible for transporting cystine and glutamate in a 1:1 ratio to maintain the intracellular amino acid balance and which is a specific reverse transporter for exchanging intracellular L-glutamic acid with extracellular L-cystine [36]. SLC7A11-mediated cystine intake is critical for the synthesis of GSH. Therefore, SLC7A11 is a key protein that negatively regulates ferroptosis. When SLC7A11 is inhibited, ferroptosis can be induced. Studies have suggested that p53 inhibits cystine uptake and makes cells sensitive to ferroptosis by inhibiting the expression of SLC7A11 [37]. Koppula et al. reported that abnormally high expression of SLC7A11 can increase GSH biosynthesis through the ingestion of cystine and inhibit the accumulation of lipid peroxidation products and ferroptosis in chronic lymphoid leukemia cells [38].

### 3.4. Mitochondrial Damage and Ferroptosis

Mitochondria are one of the most important organelles in eukaryotic cells. They are coated with two layers of membranes and are the main sites for aerobic respiration. As the “energy factory” of cells, mitochondria provide energy for cells. Mitochondria are also the main site for the generation of ROS, especially when mitochondria are damaged. Mitochondria participate in the regulation of apoptosis, autophagy, necrosis, ferroptosis, and other cell death modes. When cells enter the process of ferroptosis, the mitochondrial morphology is damaged, as reflected by a mitochondrial membrane density increase, mitochondrial volume reduction, mitochondrial crest disappearance, and outer membrane rupture [17,27,39]. In addition, the mitochondrial voltage-dependent anion-selective channel (VDAC) is a transmembrane channel for transporting ions and metabolites. The ferroptosis inducer erastin causes ferroptosis by triggering VDAC impairment, resulting in mitochondrial disorder [40]. A recent study confirmed that the mitochondrial-targeted antioxidant MitoTEMPO could block doxorubicin-induced ferroptosis in mouse cardiomyocytes [41]. Hence, mitochondrial damage has become one of the landmark events of ferroptosis.

## 4. Research Progress on Ferroptosis in Testis Dysfunction

Ferroptosis, as a new form of cell death, may be involved in male reproductive dysfunction caused by internal and external danger signals. Compared with studies on ferroptosis in other organs, there are still relatively few studies focused on ferroptosis in testicular tissues. The PubMed database was searched with “ferroptosis” and “testis” as keywords, and as of 25 December 2022, a total of 21 articles were identified. We believe that there will be more research on ferroptosis and male reproductive toxicology in the future.

### 4.1. Ferroptosis in the Testis In Vivo

Plastics manufacture-related ingredients: Plastics are an unavoidable material in our daily lives and are common components of medical equipment, packaging material, food containers, and toys. Bisphenol A is an organic chemical that is used in the manufacture of plastics. Bisphenol A, a representative endocrine disruptor, can reduce the sperm concentration in the epididymis and increase the sperm malformation rate [42]. However, the precise mechanism of male reproductive toxicity of bisphenol A has not been completely elucidated. Li et al. treated male mice with bisphenol A by gavage once daily for 45 days and found that bisphenol A caused testicular ferroptosis by diminishing GPx4 and FTH expression, boosting cyclooxygenase 2 and acyl-CoA synthetase 4 expression, and resulting in iron accumulation and mitochondrial damage in the mouse testes [43]. Ferroptosis is a new mechanism of male reproductive toxicity caused by bisphenol A. Blocking ferroptosis may be an emerging therapeutic target to antagonize the testicular toxicity of bisphenol A. Tetramethyl bisphenol A (TMBPA) is a typical bisphenol analog that is often used as a fire retardant. TMBPA impaired testosterone synthesis in Leydig cells in late puberty by causing Leydig cell ferroptosis [44]. Di-(2-ethylhexyl) phthalate (DEHP) is one of the most common plasticizers in everyday life. DEHP exposure can cause reproductive dysfunction in humans and model animals. Prepubertal DEHP exposure caused mouse testicular ferroptosis, resulting in testicular injury. Mono-2-ethylhexyl ester (MEHP), which is a major biometabolite of DEHP, can induce both Leydig and Sertoli cell ferroptosis by activating the HIF-1α/HO-1 signaling pathway [45]. Yang et al. also confirmed that DEHP decreased testosterone levels and impaired blood–testosterone barrier integrity by causing testicular ferroptosis, which was mediated by p38α–lipid ROS circulation [46].

Heavy metal toxicity: Xiong et al. treated male C57BL/6 J mice with 5 ppm Cd via drinking water in utero for 24 weeks postweaning. Cd (5 ppm) caused testicular toxicity by reducing the number of germ cells and the index of meiosis. Meanwhile, 5 ppm Cd caused iron accumulation and decreased the mRNA expression of SLC7A11 and FPN and FTH protein expression, which indicated that 5 ppm Cd-induced testicular ferroptosis may be attributed to the reduction in iron export reflected by decreasing mRNA expression of FPN [47]. Arsenite exposure caused testicular cell death, resulting in male reproductive dysfunction. However, the underlying mechanisms of arsenite-induced testicular cell death remain largely unknown. Meng et al. reported that arsenite induced testis iron accumulation, oxidative stress, and mitochondrial damage, and activated ferroptosis-related signaling pathways after 6 months in adult male mice administered 0.5, 5, or 50 mg/L arsenite through drinking water. Similarly, arsenite induced GC-2 spd cell damage by increasing iron accumulation and oxidative stress, and the toxic effect was restored using a ferroptosis inhibitor, suggesting that arsenite caused testis ferroptosis. Yet, using a ferroptosis-specific inhibitor in the drinking water to prevent or rescue the male reproductive toxicity of long-term arsenite exposure is difficult [48]. Mitochondrial damage and oxidative stress can cause other types of cell death and are not unique to ferroptosis. Arsenite can also trigger testicular apoptosis. Hence, it may be difficult to completely distinguish between testicular apoptosis and ferroptosis caused by arsenite exposure.

Drugs with male toxicity: Busulfan is a chemotherapeutic drug that affects male fertility by disrupting spermatogenesis, reducing the sperm count, and even causing the complete absence of spermatozoa (azoospermia) in semen. Zhao et al. found that busulfan treatment reduced the mouse sperm concentration and motility and decreased Gpx4 and Nrf-2 protein expression. Meanwhile, busulfan exposure decreased the expression of FPN and TfR. Inhibition of ferroptosis prevents busulfan-induced germ cell damage in the testes using the ferroptosis inhibitor ferrostatin-1 (Fer-1) or deferoxamine (DFO), demonstrating that busulfan induces testis ferroptosis [49]. Selectively inhibiting ferroptosis may represent a feasible therapeutic strategy for the treatment of busulfan-induced damage and male infertility.

Mycotoxin exposure: Zearalenone (ZEN) is the most common mycotoxin in daily life. It is produced by Fusarium fungi and is widely found in wheat, soybeans, corn, and other crops. ZEN has an estrogen-like effect, which poses a serious threat to human and animal health [50]. Li et al. established a ZEN-induced male reproductive injury model by continuous oral gavage of 30 µg/kg BW ZEN to mice for 5 weeks. ZEN blocked spermatogenesis by decreasing the motility and concentration of sperm, destroying the structure of testicular seminiferous tubules, and damaging the antioxidant defense system. ZEN also inhibited nuclear factor erythroid 2-related factor (Nrf2), SLC7A11, and GPX4 expression and induced lipid peroxidation and iron accumulation, which indicated that ZEN may have triggered testicular ferroptosis. Administration of Fer-1 relieved mouse oligozoospermia and increased SLC7A11 and GPX4 protein expression by upregulating Nrf2 expression to decrease iron accumulation. Taken together, the above results showed that ZEN induced testicular ferroptosis, resulting in spermatogenesis disorder [51]. Mitigating ferroptosis can be a novel therapeutic target for alleviating male reproductive toxicity induced by ZEN. Among the more than 300+ known mycotoxins, aflatoxin, deoxynivalenol, ochratoxin, T-2 toxin, and fumonisin are the most common, and all have reproductive toxicity. Whether the above toxins cause testicular toxicity by triggering iron death is unclear and requires further study. Ferroptosis in the testicles caused by ZEN may provide a framework for the study of the male reproductive toxicity of mycotoxins.

Reproductive damage-related model: Currently, half of all fertility problems are attributable to the male side. Oligospermia is the main symptom of males with infertility. Han et al. found that males with oligospermia have lower Nrf2 and GPX4 protein expression in sperm than healthy males. To clarify the role of Nrf2 in oligospermia, Han et al. constructed an Nrf2 gene knockout mouse model and found that Nrf2^−/−^ mice had decreased sperm concentration and motility and lower expression of GPX4 and GSH. Knockout of Nrf2 leads to spermatogenic cell ferroptosis and results in oligospermia by inhibiting the SLC7A11/GSH/GPX4 metabolic pathway, increasing intracellular iron entry, and inhibiting iron exporters. Based on the above results, targeting ferroptosis could be a treatment strategy for oligospermia with lower Nrf2 expression. In other words, Nrf2 plays a key role in regulating ferroptosis [52]. Testicular hyperthermia is one of the most important causes of spermatogenesis. Hasani et al. found that mouse scrotal hyperthermia treatment increased biomarker expression of pyroptosis (caspase-1), autophagy (Bclin-1 and Atg7), necroptosis (Mlkl) and ferroptosis (Acsl4) in testicular tissue. Combined with the previous confirmation that scrotal hyperthermia treatment could cause apoptosis, it can be concluded that scrotal hyperthermia treatment caused apoptosis, pyroptosis, autophagy, necroptosis, and ferroptosis, resulting in multiple testicular cell death modes coexisting [53]. Varicocele rats exhibit testicular lesions accompanied by testicular iron accumulation and ROS, which indicated that varicocele may cause ferroptosis. However, ferroptosis-related molecular marker levels were not significantly altered in varicocele testes, so the results do not demonstrate that testes undergo ferroptosis under varicocele conditions. The discrepancy may be attributed to varicocele testicular regional ferroptosis masking the detection of classical ferroptosis molecular markers [54]. Precisely locating the damaged area in varicocele will help to clarify the effect of ferroptosis in future research.

### 4.2. Ferroptosis of Testis-Related Cells In Vitro

Ferroptosis of Sertoli cells: PM2.5 is a common air pollutant that has reproductive toxicity and causes impairment of male fertility. Shi found that the Kyoto Encyclopedia of Genes and Genomes’ enrichment analysis of upregulated genes in PM2.5-treated TM4 cells revealed that differentially expressed genes were also enriched in the ferroptosis pathway, indicating that ferroptosis was involved in the reproductive toxicity caused by PM2.5. PM2.5 increased SLC7A11, FTH, and FTL protein expression and decreased Gpx4, suggesting that PM2.5 induced TM4 cell ferroptosis [55].

Oxygen-glucose deprivation and reoxygenation (OGD/R) in cultured cells in vitro is a common model for ischemia–reperfusion [56]. OGD/R increased TM4 cell lipid peroxidation, mitochondrial damage, iron accumulation, and death. Only the ferroptosis inhibitor Fer-1 could ameliorate the TM4 cell death caused by OGD/R, while the necrosis inhibitor necrostatin-1, autophagy inhibitor 3-MA, and apoptosis inhibitor Z-VAD-FMK had no repair effect on TM4 cell death, indicating that OGD/R caused TM4 cell ferroptosis. Iron overload may be caused by excessive iron intake, reduced iron excretion, or a combination of both. OGD/R did not alter the expression of imported and stored iron proteins (Tf, TfR, DMT1, or ferritin) but decreased FPN expression, demonstrating that OGD/R reduces iron export to induce iron overload by inhibiting FPN expression. Soon afterward, the authors found that FPN overexpression rescued OGD/R-induced ferroptosis by suppressing iron accumulation. The highlight of this study is that it reveals the reason for iron accumulation caused by OGD/R. Meanwhile, the authors also found that p38 MAPK mediates the ferroptosis induced by OGD/R, but the molecular mechanism still needs to be revealed [57].

Ferroptosis of spermatogenic cells: Pachytene spermatocytes and round spermatids were treated with the lipid peroxidation product 4-hydroxynonenal (4HNE). For pachytene spermatocytes, there was no significant difference in the level of cell death and apoptosis, but in round sperm cells, the cell death level was significantly higher than the apoptosis level, which indicated that pachytene spermatocytes and round spermatids have different sensitivities to 4HNE, and 4HNE caused nonapoptotic cell death. 4HNE-induced round spermatid death can be relieved by Fer-1 and the iron chelator DFO, demonstrating that 4HNE induces round spermatid ferroptosis. When using the ferroptosis activators erastin and RSL3 to treat pachytene spermatocytes and round spermatids, it was also found that pachytene spermatocytes exhibited no change in cell viability and round spermatid cell viability was decreased, implying differential sensitivity of germ cells to the chemical stimulation of ferroptosis [58]. The differential sensitivity of germ cells to ferroptosis may depend on the cell membrane content of PUFAs, which are easily attacked by free radicals [59].

Ferroptosis of Leydig cells: Testicular Leydig cells are mainly responsible for the synthesis and secretion of male hormones. Leydig cell death directly reduces androgen synthesis and male fertility [60]. However, no one has explored whether iron death occurs in testicular stromal cells in vitro. The authors believe that the role of ferroptosis in Leydig cell injury will receive more attention with further study of testicular ferroptosis.

## 5. Conclusions and Perspectives

Taken together, ferroptosis acts as a toxic mechanism of testicular dysfunction caused by multiple poisons. The suppression or prevention of testicular ferroptosis may be a therapeutic target/approach to alleviate male reproductive dysfunction in future research. Techniques to reduce iron accumulation and maintain testicular iron homeostasis remain to be further studied. It is still important to note that (1) cell death caused by toxic or pathogenic factors should be considered as constituting multiple cell death types, not single ferroptosis. (2) Ferroptosis may not occur in both testes at the same time; it may occur in one testis or even as a focal lesion. Further research is needed to explore how to accurately locate the site of ferroptosis (3) Is cell-specific ferroptosis induced by specific toxicants? Do only Sertoli cells, Leydig cells, or testicular spermatogenic cells undergo ferroptosis, or do they all undergo ferroptosis? Extensive research seeking to answer questions such as these is needed to elucidate the role of ferroptosis in male reproductive impairment.
